# Aquatic Fate and Ecotoxicology Effect of ZnS:Mn Quantum Dots on *Chlorella vulgaris* in Fresh Water

**DOI:** 10.3390/jox14020028

**Published:** 2024-03-26

**Authors:** Bingbing Deng, Rania Maaloul, Sophie Nowak, Yann Sivry, Claude Yéprémian, Souad Ammar, Fayna Mammeri, Roberta Brayner

**Affiliations:** 1 ITODYS, Université Paris Cité, CNRS, F-75013 Paris, Franceammarmer@univ-paris-diderot.fr (S.A.); 2 IPGP, Université Paris Cité, CNRS, F-75005 Paris, France; 3 CNRS, Molécules de Communication & Adaptation des Microorganismes MCAM, Museum National d’Histoire Naturelle, F-75005 Paris, France; claude.yepremian@mnhn.fr

**Keywords:** nanoparticles, toxicological impact, toxicity, quantum dots, behavior, dissolution, risk, fate

## Abstract

With the increasing integration of nanomaterials into daily life, the potential ecotoxicological impacts of nanoparticles (NPs) have attracted increased attention from the scientific community. This study assessed the ecotoxicity of ZnS quantum dots (QDs) doped with varying molar concentrations of Mn^2+^ on *Chlorella vulgaris*. The ZnS:Mn QDs were synthesized using the polyol method. The size of the ZnS:Mn QDs ranged from approximately 1.1 nm to 2 nm, while the aggregation size in Seine River water was 341 nm at pH 6 and 8. The presence of ZnS:Mn (10%) NPs exhibited profound toxicity to *Chlorella vulgaris*, with immediate reductions in viability (survival cells) from 71%, 60% to 51%, 52% in BG11 and Seine River water, respectively, at a concentration of 100 mg L^−1^ of ZnS:Mn (10%) NPs. Additionally, the ATP content in *Chlorella vulgaris* significantly decreased in Seine River water (by 20%) after 3 h of exposure to ZnS:Mn (10%) NPs. Concurrently, SOD activity significantly increased in Seine River water, indicating that the ZnS:Mn (10%) NPs induced ROS production and triggered an oxidative stress response in microalgae cells.

## 1. Introduction

The release of engineered materials into the environment may negatively affect living organisms in the ground, rivers, and oceans. Despite the growing prevalence of nanomaterials in various devices, research on the degradation of these materials remains scant, while numerous studies have focused on their impact on plants and potential incorporation into the food chain. With increasing apprehensions about nanotechnologies, the drive to comprehend the biological and environmental repercussions of manufactured materials is intensifying [1]. Enhancing our understanding of ecotoxicological mechanisms is crucial not only for environmental conservation but also for advancing chemistry by developing more nature-friendly protocols. For this, conducting physicochemical studies is vital for understanding the behavior of these materials, as well as their uptake and distribution within microorganisms [2,3,4]. Environmental risk assessments of nanoparticles should consider both environmental exposure (dissolution/aggregation) and hazards, such as ecotoxicity.

Among various manufactured nanomaterials, II-VI semiconducting nanocrystals stand out for their quantum confinement, enabling the tailoring of electronic and optical properties by adjusting particle size [5,6]. ZnS nanoparticles (NPs), recognized for their application in displays [7], can display different colors when doped [8,9,10,11,12,13], as dopants serve as emitting centers within the quantum-confined crystalline structure of the quantum dots (QDs) [14,15]. To generate an orange wavelength (580–590 nm), manganese is a prevalent dopant in ZnS; Mn^2+^ can replace zinc in the blende lattice as a divalent cation. The emission peak is typically attributed to the electronic transition between 4T1 and 6A1 energy levels of tetrahedral [MnS4]^6−^ molecular species [9]. However, as the exploration of the potential of ZnS nanoparticles continues, so does the imperative to understand their environmental impact and potential toxicity.

To evaluate the toxicity of ZnS quantum dots doped with Mn^2+^ and conduct ecotoxicological assays, selecting an appropriate natural setting and an aquatic model organism is crucial. Microalgae serve as excellent aquatic models due to their ability to proliferate in nutrient-rich lakes and seas, conducive to microalgae cultivation. Seine River water, known for its high mineral salt content [16,17,18], is ideal for culturing microorganisms. Microalgae are utilized in wastewater treatment [19,20] and have demonstrated proficiency in assimilating nutrients from such sources. Their ease of cultivation, relatively short growth period (<1 week), and sensitivity to pollutants [21,22,23] make them valuable for ecotoxicological studies. Certain algal species have shown remarkable abilities to eliminate some kinds of environmental pollutants (such as metal pollutants or biodegradable NPs) [24,25,26]. *Chlorella vulgaris*, recognized for its sensitivity and crucial role as a primary producer in aquatic ecosystems, has become a key organism in ecotoxicology [27,28,29]. This microalga, prevalent in diverse aquatic environments, is highly sensitive to environmental shifts. For instance, exposure to pollutants can lead to observable toxicological symptoms in *C. vulgaris* within 72–96 h, establishing it as a potent bioindicator for NP toxicity evaluation [30,31,32]. Moreover, the easy culture and maintenance of *C. vulgaris* in laboratory conditions, allowing large-scale, controlled cultivation, enhances the reproducibility of toxicological studies and minimizes logistical hurdles.

Microorganisms in natural environments are often the initial points of contact with functional engineered nanomaterials. Nonetheless, the toxicity mechanisms of NPs, such as ZnS:Mn NPs, have not yet been fully explored. While extensive research has been conducted on the toxicity of cadmium-based NPs (e.g., CdSe, CdS, CdTe) [33,34,35,36] and oxides (like ZnO NPs, TiO_2_ NPs, SiO_2_ NPs) [37,38,39], studies investigating the ecotoxicity of low-dose Mn-doped ZnS NPs to aquatic algae remain relatively scarce.

In this study, we aim to evaluate the ecotoxicity of ZnS:Mn nanoparticles (synthesized via the polyol process) when interacting with *Chlorella vulgaris* in Seine River water [16]. The ecotoxic effects of ZnS NPs with varying Mn^2+^ dopant concentrations on microalgae were investigated. Specifically, we examined the impact of ZnS:Mn NPs on mitochondrial activity (Adenosine TriPhosphate (ATP) content), superoxide dismutase (SOD) activity, photosynthetic activity, and microalgae viability. Notably, Mn^2+^ can dissolve in the aquatic system and may pose toxicity to *C. vulgaris*. The dissolved Mn^2+^ or ZnS:Mn NPs, once internalized by algal cells, could affect the functionality of chloroplasts and mitochondria. This is primarily achieved through the induction of reactive oxygen species (ROS), potentially leading to cell death.

## 2. Materials and Methods

### 2.1. Synthesis of Mn-Doped ZnS Quantum Dots Using the Polyol Method

All chemicals are of analytical grade and were used without any further purification. Manganese acetate tetrahydrate (≥99%, Mn(CH_3_COO)_2_·4H_2_O), trioctylphosphine oxide (TOPO, (CH_3_(CH_2_)_7_)_3_PO), and diethylene glycol (DEG) (OH(CH_2_)_2_O(CH_2_)_2_OH) were purchased from Sigma-Aldrich, Saint-Quentin Fallavier, France; thiourea (99%, SC(NH_2_)_2_) was purchased from Alfa Aesar, Paris, France; and zinc acetate dihydrate (98 + %, Zn(CH_3_COO)_2_·2H_2_O) was purchased from Acros Organics, Illkirch, France.

Zinc acetate dihydrate (Zn(CH_3_COO)_2_·2H_2_O, 87.8 mg) and manganese acetate tetrahydrate (Mn(CH_3_COO)_2_·4H_2_O, 1.0 mg, 4.1 mg, 8.5 mg and 24.5 mg) were mixed in a three-neck flask with 80 mL of diethylene glycol (DEG) to synthesize ZnS:Mn (0.5%), ZnS:Mn (2.0%), ZnS:Mn (4.0%), and ZnS:Mn (10%) in presence of thiourea (38.8 mg) and TOPO (193.3 mg). All the reagents were dissolved in DEG with 20 min of sonication, and then the solutions were heated to 180 °C for 30 min; after that, ZnS:Mn (0.5%), ZnS:Mn (2.0%), ZnS:Mn (4.0%); ZnS:Mn (10%) NPs were recovered after 3 cycles of centrifugation (at 22,000× *g* rpm) and washing with ethanol, and then dried at 60 °C overnight.

### 2.2. Characterization of ZnS:Mn QDs

The crystalline structure of the synthesized ZnS:Mn NPs was analyzed using XRD with a X’Pert Pro Pananalytical diffractometer (Panalytical, Almelo, The Netherlands), employing Co Kα radiation over a range of 10–80° (2θ) with a scan step of 0.016°. Peak indexing was executed utilizing Highscore Plus software (version 5.2, Panalytical, The Netherlands) and the ICSD-Panalytical Database. Cell parameters and coherent diffraction domain size were determined using MAUD software (version 2.92, Trento, Italy) [40], based on the Rietveld method.

The surface chemical composition analysis of the NPs was performed through XPS using a Thermo VG ESCALAB 250 instrument (ThermoFisher Scientific, East-Grinstead, UK) equipped with a micro-focused monochromatic Al Kα X-ray source (1486.6 eV) and a magnetic lens. The X-ray spot size was 500 µm (15 kV, 150 W). Spectra were collected in constant analyzer energy mode with pass energies of 100 and 40 eV for survey spectra and high-resolution regions, respectively. Data acquisition and processing were conducted using “Avantage” software (version 4.67). The C1s peak at 285 eV, attributed to adventitious contamination, served as a reference for binding energy calibration and charge correction.

Mn, Zn, and S ratios were determined by XRF spectrometry (Panalytical, Almelo, The Netherlands). Nanopowders were dispersed in demineralized water, and 20 µL of aliquots was deposited on clean polycarbonate membranes and dried. The membranes were then analyzed using an Epsilon 3XL (Panalytical) XRF spectrometer equipped with a silver X-ray tube. Operating conditions varied: 20 kV and 15 µA for Mn, 50 kV and 6 µA for Zn, and 10 kV and 30 µA for S. A certified solution of Mn, Zn, and S (Inorganic Ventures 1 g/L) was utilized for calibration under identical conditions, within a range from 0 to 30 µg.

The morphology of the NPs was assessed by transmission electron microscopy (TEM) using a JEOL-100 CX II microscope (JEOL, Tokyo, Japan) at 100 kV. The mean diameter and standard deviation were derived from an image analysis of approximately 250 particles, conducted using ImageJ software (Version 1.53).

Dynamic light scattering (DLS) and zetametry were utilized to assess the size distribution and surface charge of the NPs using a Zetasizer (Spectris plc., London, UK) from Malvern. The ζ potential was measured at room temperature, beginning with their aqueous solution (1 g L^−1^) after vigorous sonication for 10 min. The concentration of these solutions was 0.1 mg mL^−1^, with pH levels of 2, 4, 6, 8, and 10, respectively.

The dissolution concentrations of Mn and Zn were quantified via inductively coupled plasma–atomic emission spectroscopy (ICP-AES) (ICAP 6200 Thermo Fisher, Thermo Fisher Scientific, Waltham, MA, USA). Detection and quantification limits were 0.20 ppb and 0.67 ppb for Zn, and 0.02 ppb and 0.07 ppb for Mn, respectively. The standard deviation associated with these measurements was less than 5%. The concentration of solutions used for ICP-AES was 0.01 mg mL^−1^.

### 2.3. Chlorella vulgaris Culture

*C. vulgaris* were sourced from the Museum National d’Histoire Naturelle (MNHN) Culture Collection. *C. vulgaris*, a planktonic eukaryotic single-cell green alga, was cultivated in 75 cm^2^ cell culture flasks purchased from Thermo Fisher in (i) sterile BG11 medium (pH 7.30) and (ii) Seine River water (SRW, pH 8.11). Then, 15 mL of *C. vulgaris* culture was mixed with 45 mL of various media, and then all cultures were maintained at a controlled temperature of 25 °C and a daily light cycle of 16 h of luminosity (50–80 μmol m^−2^ s^−1^ photosynthetic photon flux, PPF) in an ambient CO_2_ environment. The live cell concentration in these media (BG11, SRW) was approximately 8.84 × 10^6^ cells mL^−1^ and 5.59 × 10^6^ cells mL^−1^, before commencing the experiments.

Seine River water was taken from the river close to Université Paris Diderot, France (GPS: 48.828335° N, 2.385351° E), immediately filtered with a 0.22 μm acetate membrane (Millipore)(Sigma-Aldrich, Saint-Quentin Fallavier, France), autoclaved (121 °C for 20 min), and then stored at 4 °C in a fridge. The average chemical composition of Seine River water is shown in Table 1.

### 2.4. Ecotoxicity Assessments

Survival viability was assessed using the Cellometer Auto T4 (Nexcelom, Lawrence, KS, USA). SOD (superoxide dismutase) enzymatic activity was measured using the 19,160 SOD Determination Kit from Sigma-Aldrich (Saint-Quentin-Fallavier, France). SOD activity was colorimetrically evaluated through a kinetic method and readings were taken at 450 nm using an Envision Multilabel Plate Reader (Perkin-Elmer, Waltham, MA, USA).

ATP levels in the samples were detected using the luciferase–luciferin enzymatic assay kit BacTiter-Glo™ from Progema (Charbonnières-les-Bains, France). This kit facilitates the release of ATP from algal cells through cell lysis, negating the need for cell washing or medium removal. The reagent can be directly added to the microplate well. ATP quantitation was performed using an Envision Multilabel Plate Reader (Perkin-Elmer, Waltham, MA, USA) equipped with a luminescent optical filter. All assays were conducted in triplicate, and the test reagents used in the ecotoxicity assessments are also from the same batch.

Biomass transmission electron microscopy (TEM) studies were carried out using a Hitachi H-700 (Tokyo, Japan) at 80 kV, equipped with a Hamatsu camera (Massy, France). The microalgae were initially fixed with a mixture containing 2% glutaraldehyde and picric acid in a Sorengën phosphate buffer (0.1 M, pH 7.4). Cells were contrasted with 0.5% osmium tetroxide. Dehydration was then achieved through a series of ethanol baths, after which the samples were processed for flat embedding in Supper resin. Ultrathin sections of the samples in resin were then produced using a Reicherd E Young Ultracut ultramicrotome (Leica, Wetzlar, Germany).

### 2.5. Statistical Analysis

Statistical analyses were performed using SPSS v.26.0. For comparisons among control and treatments, one-way analysis of variance (ANOVA) test followed by Tukey’s post hoc test was conducted for the variables that met the criteria of normality (Shapiro–Wilk test, *p* > 0.05), homoscedasticity (variance homogeneity, Levene test, *p* > 0.05) and independence (Durbin Watson test, *p*~2.0). For the variables that did violate the assumptions for ANOVA validity, a non-parametric Kruskal–Wallis test was conducted. Differences between groups were considered statistically significant when *p* ≤ 0.05 and marginally significant when 0.05 < *p* ≤ 0.1.

## 3. Results and Discussion

### 3.1. Structural Characterization of ZnS:Mn NPs

**Figure 1:**
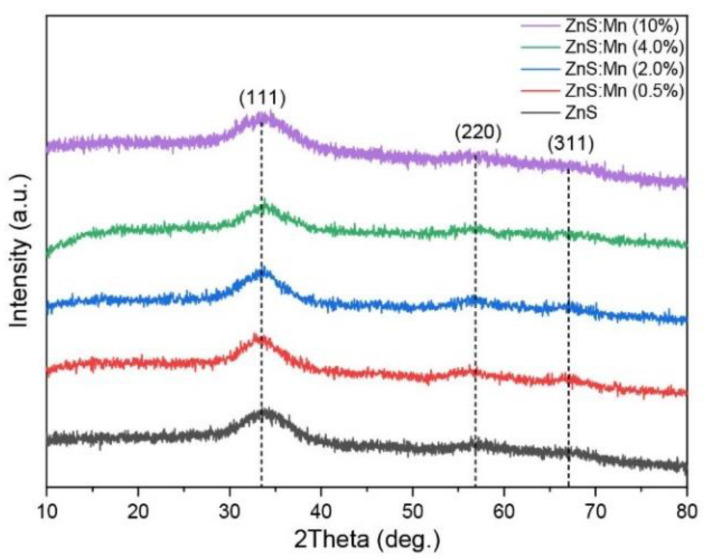
XRD patterns of all the produced ZnS:Mn NPs.

X-ray diffraction (XRD) patterns of the ZnS:Mn NPs are illustrated in Figure 1. The diffraction peaks observed at 33°, 56°, and 67° correspond to the (111), (220), and (311) crystalline planes of the cubic ZnS phase (COD no. 5000088), indicating the presence of a singular phase, and thus the high purity of the ZnS:Mn NPs, despite their small size. The breadth of the peaks suggests the nanocrystalline nature of the samples [41]. The crystal sizes were computationally determined through Rietveld refinements using MAUD software (Materials Analysis Using Diffraction, Version 2.998), ranging between 1.1 and 1.5 nm. The average crystalline size of these ZnS NPs was further estimated using the full width at half maximum (FWHM) of the diffraction peaks and applying the Debye–Scherrer formula [41,42]:

$$D=\frac{k\lambda }{\beta cos\theta }$$

where *D* is the average size of the particles, *k* is the particles’ shape factor (0.89), *λ* is the X-ray wavelength (0.17889 nm), *β* is the FWHM of the diffraction peak, *θ* is the diffraction Bragg angle. The calculated average crystalline sizes of ZnS:Mn (0.5%, 2.0%, 4.0%, and 10%) NPs ranged from 2.3 nm to 2.5 nm. This range aligns well with the average diameter deduced from the TEM analysis (2.3 ± 0.5 nm), as shown in Figure 4. It is worth noting that the Debye–Scherrer formula was used to calculate size, and the TEM statistical size was slightly larger than the actual crystal size, which can be attributed to the 2θ measurement boundary and other factors.

Table 2 gathers the elemental compositions of ZnS:Mn nanoparticles deduced from XRF analysis: the Mn content in the synthesized NPs increased from 0.002 to 0.168, while increasing the theoretical molar concentration of Mn (Mn/(Mn + Zn)_exp_ from 0.005 to 0.100, although this does not match well with the expected molar concentration of Mn.

**Figure 2:**
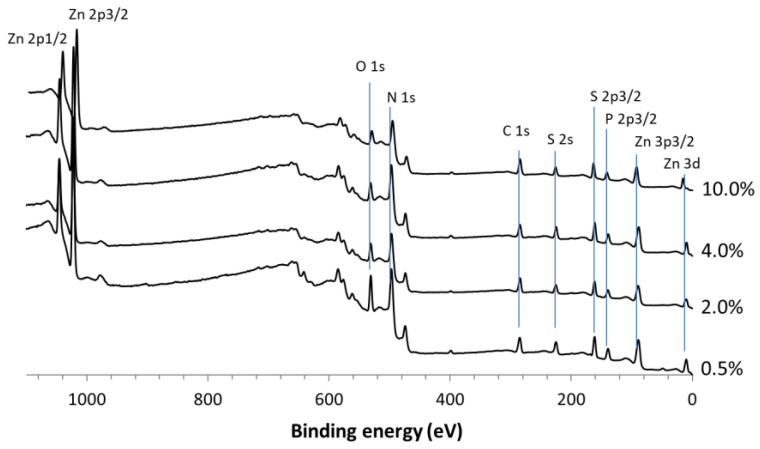
XPS survey spectra of ZnS:Mn (0.5%, 2.0%, 4.0% and 10%) NPs, the spectra were calibrated by setting the main C 1s at 285 eV.

The surface chemical composition of the NPs was examined through an XPS analysis. The survey spectra of ZnS:Mn (0.5%, 2.0%, 4.0%, and 10%) NPs are displayed in Figure 2. The binding energies for Zn 2p_1/2_ and Zn 2p_3/2_ were identified at 1045.8 eV and 1022.9 eV, respectively, confirming the presence of Zn solely in the Zn^2+^ state. The S 2p peak at 161.4 eV is characteristic of the S^2−^ species [43,44]. Semi-quantitative XPS data revealed atomic Zn/S ratios of 1:0.9, 1:1, 1:1, and 1:0.9 for the 0.5%, 2.0%, 4.0%, and 10% samples, respectively, approximating the ideal 1:1 ratio for each sample. Peaks indicative of C and O adventitious contaminations were also observed in all spectra. The slight deviation in the Zn/S ratio to 0.9 might suggest the possible oxidation of the NPs. Nevertheless, the O 1s peak at 532.0 eV is not indicative of O atoms in a ZnO crystalline system, as signals from ZnO would manifest at 530 eV. Thus, the O 1s peak at 532 eV is likely attributable to chemisorbed oxygen species [43].

**Figure 3:**
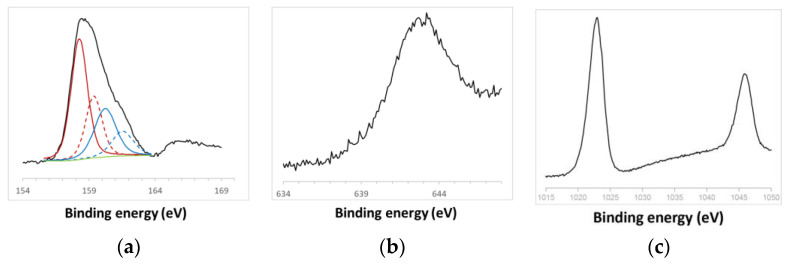
High-resolution spectra of (**a**) S 2p, (**b**) Mn 2p and (**c**) Zn 2p signals recorded on ZnS:Mn NPs with 10% Mn.

The high-resolution spectra of S 2p, Mn 2p, and Zn 2p elements are depicted in Figure 3. The spin–orbit splitting peaks’ intensity ratio for S 2p_3/2_ and S 2p_1/2_ was approximately 2:1, consistent with the presence of Zn^2+^ exclusively bonded to S atoms [45]. The asymmetric spectra of S 2p for ZnS:Mn (10%) NPs could be deconvoluted into two components, each with two peaks corresponding to S 2p_3/2_ and S 2p_1/2_. The binding energies at 158–159 eV stemmed from S^2−^ within the ZnS structure, while the subpeak around 160–161 eV might be attributable to surface defects of S–S species in the ZnS shell layer, as previously reported for ZnS nanorods [46]. Additionally, a less intense S 2p peak at 167.9 eV, indicative of the oxidized SO_4_^2−^ form, was observed [47], suggesting the slight and negligible surface oxidation of ZnS:Mn NPs. As the Mn content increased, the spectra became progressively less noisy [48,49,50]. The Mn 2p_3/2_ peak, centered at approximately 642 eV, corresponded to Mn^2+^ [51]. The binding energies for Zn 2p_1/2_ and Zn 2p_3/2_ were identified at 1045.8 eV and 1022.9 eV, respectively, affirming Zn in the Zn^2+^ state. Semi-quantitative XPS data presented atomic Mn/S ratios of 0.02:1, 0.03:1, 0.04:1, and 0.08:1 for the 0.5%, 2.0%, 4.0%, and 10% samples, respectively, closely aligning with the ideal ratios for each sample.

**Figure 4:**
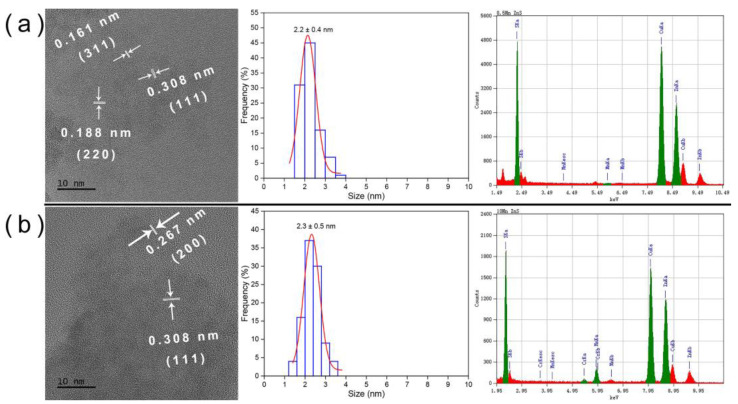
TEM image, size distribution and EDS of (**a**) 0.5% ZnS:Mn and (**b**) 10% ZnS:Mn. (2.0% and 4.0% Mn doped ZnS are shown in Figure S1).

Transmission electron microscopy (TEM) images, as shown in Figure 4, Figures S1 and S2, displayed different molar concentrations of Mn-doped ZnS NPs. The TEM images provided lattice information and confirmed the blende structure of crystallized ZnS:Mn (0.5%, 2.0%, 4.0%, and 10%). Statistical analysis of the NP size distribution indicated an average size of approximately 2.3 ± 0.5 nm. Furthermore, an energy-dispersive X-ray (EDX) analysis revealed the primary constituents of these NPs to be Mn, Zn, and S elements. Cu was identified as a component of the grid, and Cr originated from the TEM machine, suggesting the absence of impurities in the ZnS:Mn NPs.

**Figure 5:**
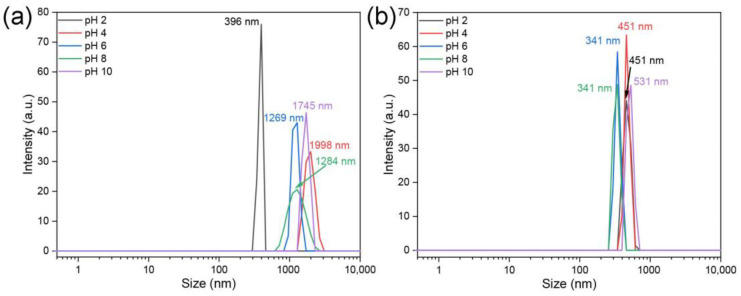
Size of ZnS:Mn (10%) NPs in (**a**) BG11 and (**b**) Seine River water: (0.5%, 2.0% and 4.0% Mn doped ZnS NPs are shown in Figure S3).

The size and colloidal stability of the NPs were assessed using dynamic light scattering (DLS) in BG11 and Seine River water across varying pH levels (Figure 5 and Figure S3). Irrespective of the medium and pH, the NPs aggregated to varying extents. In BG11 (or fresh) water at pH 2, all NPs formed smaller aggregates with colloidal sizes of around 340 nm for ZnS:Mn (0.5%) and ZnS:Mn (4.0%) NPs, and 460 nm and 400 nm for ZnS:Mn (2.0%) and ZnS:Mn (10%), respectively. In more basic conditions (pH 8), the colloidal size of almost all samples exceeded 1000 nm, except for ZnS:Mn (4.0%) which remained below 340 nm. In Seine River water, the colloids were relatively stable, exhibiting sizes between 340 nm and 530 nm across different pH levels, except for ZnS:Mn (4.0%) NPs, which formed aggregates of 220 nm at pH 4 and larger than 2000 nm at pH 10. Overall, NP aggregate sizes were more stable in BG11 under acidic conditions. In Seine River water, colloids maintained stability (300 to 500 nm) regardless of the medium being acidic or alkaline, with the exception of ZnS:Mn (4.0%) NPs, which tended to aggregate more readily when the pH varied from 6 to 8. Additionally, the size of ZnS:Mn (10%) NPs in Milli-Q water was 440 nm (at pH 6.89, Figure S4). It is important to note that the pH of *Chlorella vulgaris* cultures in different water systems ranges from 7.4 to 8.7.

**Figure 6:**
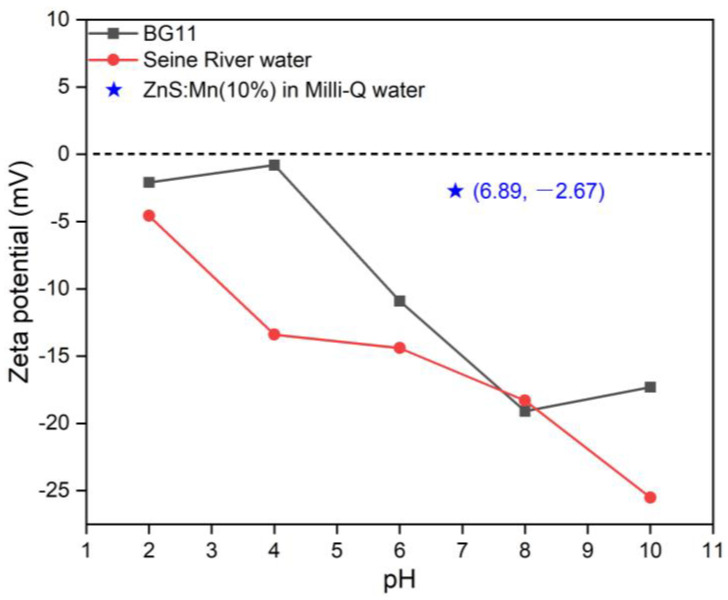
Zeta potential of ZnS:Mn (10%) NPs in different water systems: (0.5%, 2.0% and 4.0% Mn doped ZnS NPs are shown in Figure S3).

Figure 6 and Figure S5 illustrate the zeta potential measurements of all produced NPs in these two water systems across varying pH levels. It was noted that the zeta potential values of the NPs in fresh water (BG11) and Seine River water were approximately or greater than 20 in alkaline conditions (pH: 8 to 10), indicating the relative stability of the NPs in alkaline media in both BG11 and Seine River water. In all aquatic environments, NPs were negatively charged when pH ranged from 6 to 8. Furthermore, the zeta potential value of ZnS:Mn (10%) NPs in Milli-Q water was recorded as −2.67, denoting instability in pure water, while indicating relative stability in mildly acidic or alkaline environments. Given that the zeta potential of algal cells is 8.82, nanoparticles and algae would likely repel each other electrostatically, impeding the internalization of NPs by the cells [52,53].

**Figure 7:**
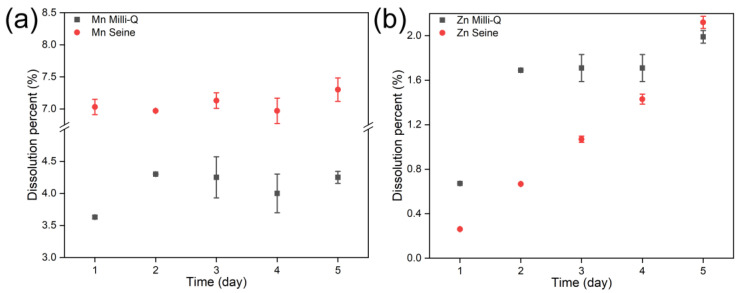
Dissolution of ZnS:Mn (10%) NPs in different water systems: monitoring of (**a**) Mn^2+^ and (**b**) Zn^2+^ concentrations in different water systems.

Despite the tendency of NPs to aggregate in water across pH levels, dissolution is possible. Hence, the dissolution of ZnS:Mn (10%) was monitored by ICP-AES. Figure 7 displays the concentrations of Mn^2+^ and Zn^2+^ in these two water systems after dissolving ZnS:Mn (10%) NPs. Mn^2+^ dissolution was generally higher in Seine River water compared to Milli-Q water. In Milli-Q water, peak Mn^2+^ dissolution occurred on the 2nd day, whereas it reached its zenith on the 5th day in Seine River water. Interestingly, Zn^2+^ dissolution exhibited distinct behavior: in Seine River water and Milli-Q water, Zn^2+^ dissolution persisted and intensified over time.

### 3.2. Assessment of the Toxicity of ZnS:Mn Nanoparticles

The toxicity of ZnS:Mn NPs to *Chlorella vulgaris* may be influenced by various factors, including composition, nanoparticle size, concentration, surface coating, and exposure duration. Unlike their bulk counterparts, nanomaterials like ZnS:Mn NPs have the potential to distinctively interact with living organisms due to their unique nanoscale properties. The exposure of *C. vulgaris* to nanoparticles can affect several biological processes, such as cellular uptake, oxidative stress, and alterations in biochemical pathways. These interactions may lead to consequences such as inhibited growth, reduced photosynthesis, lower cell viability, and altered metabolic functions.

To discern whether toxicity originated from Mn^2+^ ions or the NPs themselves, Mn(CH_3_COO)_2_·4H_2_O salt, pure ZnS NPs, and ZnS:Mn NPs were introduced into two different culture media of *C. vulgaris*: BG11 and Seine River water (SRW). After one week, varying concentrations of NPs (20 mg L^−1^, 50 mg L^−1^, and 100 mg L^−1^) were administered to the aquatic media, with each medium represented in four culture flasks (three experimental groups and one control group). Relative toxicity indicators, such as cell viability, superoxide dismutase (SOD) activity, and mitochondrial activity, were monitored for five consecutive days following exposure to the NP environment. This approach facilitated the evaluation of NP toxicity by comparison with the control group.

#### 3.2.1. Toxicity of ZnS NPs

**Figure 8:**
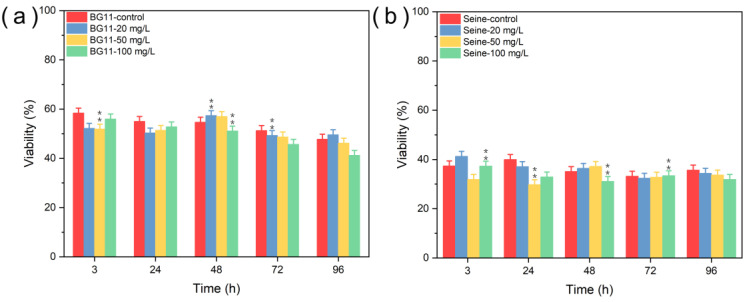
Viability (survival) tests of *Chlorella vulgaris* in (**a**) BG11, (**b**) SRW culture media contacted with pure ZnS NPs. Values represent mean ± standard deviation (n = 3). Differences among groups are indicated by an asterisk (marginally significant, 0.05 < *p* ≤ 0.1) or double asterisk (significant, *p* ≤ 0.05). Double asterisk indicates that the difference between groups is statistically significant.

Figure 8 illustrates the viability of *Chlorella vulgaris* in different aqueous media when exposed to ZnS NPs. In both BG11 and SRW media, a notable decline in viability (about 3%) was observed from the initial day of exposure, persisting until the test’s conclusion, relative to the control group. However, a recovery in viability was noted 48 h later at lower NP concentrations (20 mg L^−1^ and 50 mg L^−1^). In Seine River water, viability decreased during the initial days of testing at higher NP concentrations (50 mg L^−1^ and 100 mg L^−1^), with the exception of the fourth day. Moreover, algae exhibited a substantially reduced viability (below 40%) in SRW media, even in the control group, and this reduction may be attributed either to the media’s influence on cell membrane protein expression [54] or to the significant aggregation of ZnS NPs on the algal surface, potentially disrupting cellular membrane transport functions and consequently reducing the nutrient availability essential for algal growth. Thus, it can be inferred that ZnS NPs exert a weak toxic impact on *C. vulgaris*, corroborating findings from previous studies [55,56]. As expected, higher NP concentrations manifested greater toxicity compared to lower concentrations.

#### 3.2.2. Toxicity of Mn^2+^

The toxicity of Mn^2+^ was investigated by introducing Mn^2+^ solutions at concentrations of 20 mg L^−1^, 50 mg L^−1^, and 100 mg L^−1^. Figure S6 depicts the state of *Chlorella vulgaris* cultures in the presence of Mn^2+^ ions 96 h post-introduction. The cultures in Seine River water containing Mn^2+^ ions exhibited a notably darker color compared to the control groups, especially in the culture medium containing high concentrations of Mn^2+^ (100 mg L^−1^).

**Figure 9:**
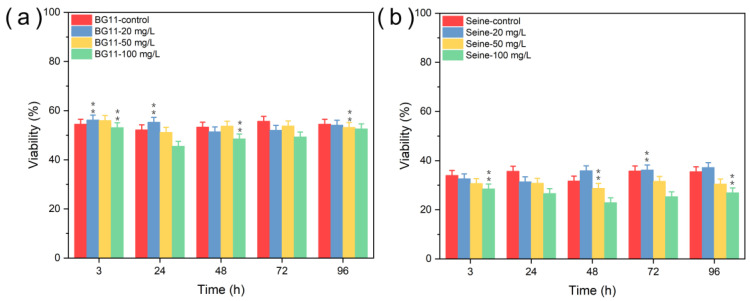
Viability (survival) tests of *Chlorella vulgaris* in (**a**) BG11 and (**b**) SRW culture media contacted with Mn^2+^ salt. Values represent mean± standard deviation (n = 3). Differences among groups are indicated by asterisk (marginally significant, 0.05 < *p* ≤ 0.1) or double asterisk (significant, *p* ≤ 0.05). Double asterisk indicates that the difference between groups is statistically significant.

Figure 9 presents the viability of *C. vulgaris*: in BG11 media, viability was nearly stable at Mn^2+^ concentrations up to 100 mg L^−1^. However, at 100 mg L^−1^ of Mn^2+^, a significant reduction in viability was noted during the first four days of testing. In SRW media, viability decreased variably, likely due to nutrient deficiencies, such as nitrates, phosphates, or carbon sources (e.g., glucose), which are lacking in Seine River water [38]. As a result, the physiological condition of the algae deteriorated owing to nutrient scarcity, making them more susceptible to the adverse conditions of the water or the potentially toxic effects of Mn^2+^ ions. In contrast, algae in BG11 water, supplied with adequate nutrients, exhibited a markedly different response. Thus, high concentrations of Mn^2+^ ions (e.g., 100 mg L^−1^) were profoundly toxic to *C. vulgaris* across all culture media, with the toxicity being particularly pronounced in SRW medium.

#### 3.2.3. Toxicity of ZnS:Mn (10%) NPs

The doping of nanoparticles with transition metal cations, such as manganese, can alter their toxicity and interactions with living organisms compared to their undoped counterparts. Specifically, a transition metal cation like Mn^2+^, not originally present in the base nanoparticles, is incorporated into the crystal lattice during the synthesis process. Consequently, the toxicity of ZnS:Mn (10%) NPs was evaluated (results detailing the toxicity of ZnS:Mn (0.5%), ZnS:Mn (2.0%), and ZnS:Mn (4.0%) are illustrated in Figures S7–S12).

**Figure 10:**
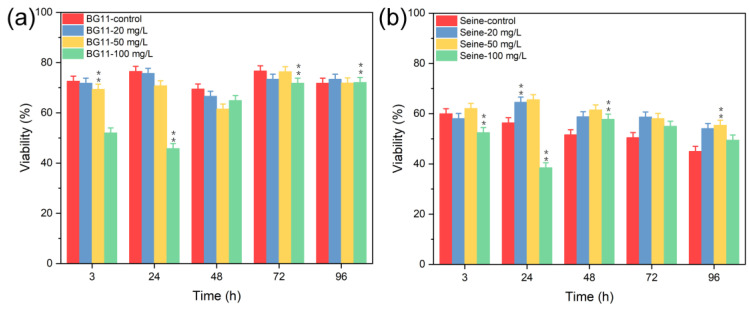
Viability (survival) tests of *Chlorella vulgaris* in (**a**) BG11 and (**b**) SRW culture media contacted with ZnS:Mn (10%) NPs. Values represent mean± standard deviation (n = 3). Differences among groups are indicated by an asterisk (marginally significant, 0.05 < *p* ≤ 0.1) or double asterisk (significant, *p* ≤ 0.05). Double asterisk indicates that the difference between groups is statistically significant.

Figure 10 shows a significant decrease in *Chlorella vulgaris* viability immediately following exposure to 100 mg L^−1^ of ZnS:Mn (10%) NPs in these aqueous media; the viability was approximately 50% in BG11 and SRW. At a 50 mg L^−1^ NP concentration, the viability in BG11 significantly decreased during the initial three days of testing before gradually returning to normal levels [57]. At a concentration of 20 mg L^−1^, viability also slightly declined, notably in BG11 from 3 h to 72 h. These observations lead to the conclusion that a concentration of 100 mg L^−1^ of ZnS:Mn (10%) NPs poses a significant threat to the survival of *C. vulgaris*, and even lower concentrations of these NPs manifest toxicity to *C. vulgaris* in BG11. Interestingly, while viability decreased, photosynthetic activity did not show a corresponding decline (as seen in Figure S13). This resilience might be attributed to a protective mechanism involving carotenoids, a type of non-enzymatic antioxidants, which possibly act as singlet oxygen quenchers, thus safeguarding photosynthetic machinery [58].

**Figure 11:**
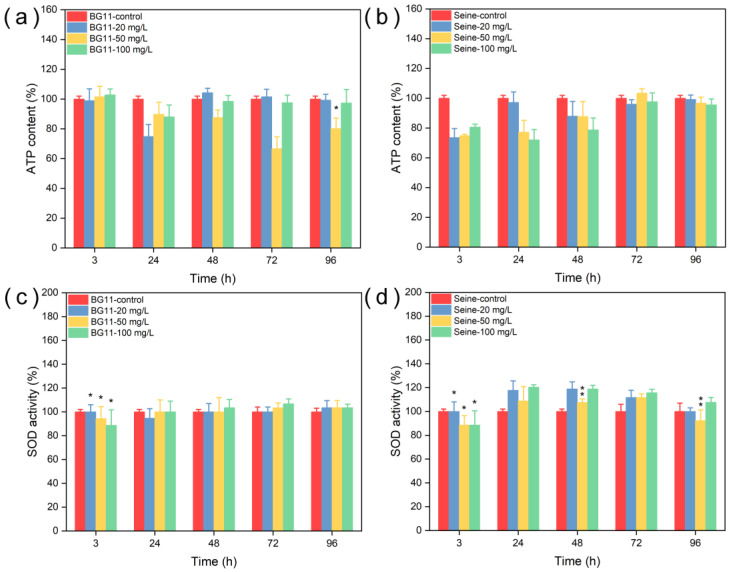
ATP (**a**,**b**) and SOD (**c**,**d**) tests of *Chlorella vulgaris* in different culture media contacted with ZnS: Mn (10%) NPs. Values represent mean ± standard deviation (n = 3). Differences among groups are indicated by asterisk (marginally significant, 0.05 < *p* ≤ 0.1) or double asterisk (significant, *p* ≤ 0.05).

The impact of NPs on the mitochondrial activity of algal cells was gauged through the intracellular adenosine triphosphate (ATP) content, as depicted in Figure 11. In BG11, ATP content experienced a decline after 24 h of exposure compared to the control group, and this effect was more pronounced at a concentration of 50 mg L^−1^ after 72 h. While mitochondrial activity significantly decreased post 3 h of exposure in SRW, the detrimental impact on mitochondria persisted over an extended period. The reduction in ATP content indicates a decrease in mitochondrial activity, signifying that ZnS:Mn NPs (10%) disrupted the energy metabolism of the algae. Superoxide dismutase (SOD) activity, as shown in Figure 11, exhibited a marginal increase in BG11 after 48 h, particularly at a concentration of 100 mg L^−1^ of NPs. Moreover, SOD activity significantly rose in SRW after 24 h, and in the group with 100 mg L^−1^ of NPs, the SOD activity has always been at a high level. These observations suggest that NPs triggered the production of reactive oxygen species (ROS), especially in SRW, impacting mitochondrial activity. This led to a notable reduction in ATP production, which consequently influenced photosynthetic activity and cell viability.

**Figure 12:**
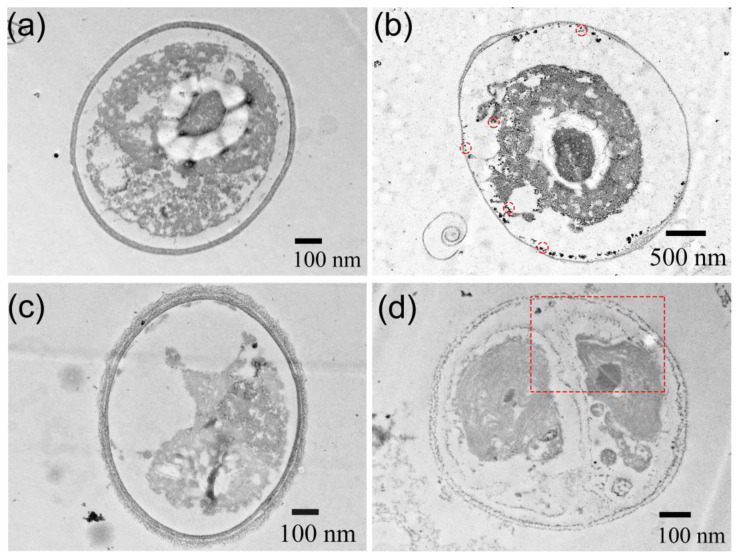
TEM images of thin sections of *Chlorella vulgaris* in different media: (**a**,**c**) in BG11 and in SRW; (**b**,**d**) show the thin sections of *Chlorella vulgaris* after exposure to 100 mg L^−1^ of ZnS:Mn (10%) NPs. Areas with red dotted lines mean that there are many NPs in the algal cell.

Thin-section TEM images, presented in Figure 12 and Figure S14, showcase *Chlorella vulgaris* cells with and without exposure to ZnS:Mn (10%) NPs. In the absence of NPs, the integrity of the cell wall and the clarity of the cytoplasm are evident across all aqueous media. Conversely, the presence of ZnS:Mn (10%) NPs resulted in noticeable damage to the cell wall and/or cytoplasm. In BG11, dark, circular, and spherical NP aggregates are visibly attached to the cell wall, with a pronounced separation between the cytoplasm and cell wall. In SRW, smaller NPs were also observed within the cytoplasm, accompanied by the breakdown of the cell wall. The images further reveal numerous aggregated NPs within the algal cell, the disappearance of the cytoplasm, and the presence of NPs (circled in the image) outside the algal cell. Although the precise mechanisms of algal cell NP uptake remain unconfirmed, the following mechanisms are proposed: direct contact of NPs with the cell membrane, leading to interactions with lipid molecule groups and subsequent pore formation, and the internalization of NPs by cells through endocytosis [59]. Regardless of the mechanism, NPs inside algal cells can cause the destruction of the cytoplasm and cell wall, posing a lethal threat to the cells.

In this study, the synthesis of ZnS and ZnS:Mn NPs with Mn^2+^ concentrations ranging from 0.5% to 10% was detailed. All synthesized particles exhibited uniform diameters. DLS analysis revealed that these NPs tend to aggregate variably between pH 6 and 8, with the degree of aggregation dependent on the specific aqueous medium. The NPs demonstrated better dispersion in SRW compared to freshwater. Zeta potential measurements indicated good stability in freshwater and Seine River water, although in all environments, the NPs were negatively charged, posing challenges for internalization from the outset.

Focusing on particles with 10% Mn^2+^, an ICP-AES analysis indicated the substantial dissolution of Mn^2+^ ions in Seine River water, whereas Zn^2+^ ions showed a higher dissolution rate in freshwater and Seine River water. Toxicity assays revealed that ZnS NPs had a poor toxicity to *Chlorella vulgaris* across all water types. Observations based on the color of solutions suggested that Mn^2+^ ions exhibited toxicity at 100 mg L^−1^ concentrations, with a milder toxicity noted in Seine River water. Similarly, ZnS:Mn NPs demonstrated pronounced toxicity at concentrations around 100 mg L^−1^, and a partial toxic effect on *Chlorella vulgaris* may stem from ZnS:Mn NPs dissolving Mn^2+^ in the culture medium; with some dissolved ions (presumably Zn^2+^ ions) potentially serving as nutrients in Seine River water.

A marked reduction in ATP content was noted in freshwater and Seine River water, indicating a significant disruption of algal energy metabolism by ZnS:Mn (10%) NPs. However, a subsequent increase in ATP content in SRW media suggests that algae may adapt to the medium and restore ATP production.

SOD content almost remained stable in freshwater, only slightly increased 48 h later, and showed a significant increase in SRW media before stabilizing. This suggests that NPs induced ROS production, impairing mitochondrial activity and consequently reducing ATP production. This, in turn, affected photosynthetic activity and cell viability. However, with the enhancement of the SOD activity in the algae cells, ROS productions are gradually cleared, which alleviates the oxidative stress response in the algae cells. Subsequently, ATP levels slowly recovered, and cell viability almost returns to normal levels after 96 h. The growth of the algal community in the medium appears to be recovered.

Hence, ZnS:Mn NPs activate an antioxidant defense system within the algal cell, which may not always successfully counteract these assaults. Subcellular organelle degradation, as observed in TEM images, and oxidative stress are primary outcomes of NP toxicity. This toxicity can be ascribed to several factors including the size of the aggregates, their composition, and their ion dissolution stability. Investigations on manganese salt indicate that Mn^2+^ ions must reach very high concentrations before transitioning from a nutrient to a toxin. Conversely, ZnS NPs were identified as having a poor toxicity. Additionally, the natural organic matter present in aquatic environments may modulate NP toxicity due to its heterogeneous composition and numerous functional groups [60], potentially altering NP behavior in aquatic settings and mitigating the toxicity of ZnS:Mn NPs [61]. The interplay between natural organic matter in natural water environments and the toxicity of ZnS:Mn NPs warrants further exploration.

In conclusion, nanoparticles can be considered toxic to *Chlorella vulgaris* via an oxidative stress mechanism. The high surface-to-volume ratio of NPs enables them to encircle the algal cell, despite general aggregation, thereby reducing the nutrient exchange surface of algal cells, in addition to facilitating NP internalization.

## 4. Conclusions

In this study, the synthesis of ZnS:Mn NPs (0.5%, 2%, 4%, and 10%) via the polyol method was detailed. The NPs exhibited a zinc blende structure with relatively small sizes, ranging from approximately 1.1 nm to 2 nm. When introduced into aqueous systems, these NPs were prone to aggregation, potentially impacting nutrient uptake by *Chlorella vulgaris*. Furthermore, these NPs influenced the SOD activity, ATP content, and viability of *C. vulgaris*, and could even lead to algal cell death. Notably, in Seine River water, a significant reduction in ATP content and an increase in SOD activity were observed. This suggests that NPs can create an oxidative stress environment, affecting mitochondrial activity and resulting in cell death. Additionally, in BG11 medium, the biological activity of *C. vulgaris* was also affected, with a substantial decline in viability was observed at a ZnS:Mn (10%) NP concentration of 100 mg L^−1^.

The potential toxicity of manufactured nanoparticles is multifaceted and is compounded by the complexity and diversity of biological environments, which can result in variable responses among algal cultures. Fortunately, *C. vulgaris* demonstrated consistent behavior, allowing us to conclude that while the toxicity of manganese-doped ZnS nanoparticles is evident, it is less severe than the toxicity induced by Mn^2+^ ion concentrations of 100 mg L^−1^. Consequently, the integration of these particles into miniaturized devices can be considered with a degree of confidence.

## Figures and Tables

**Table 1:** Chemical composition of Seine River water [16].

×10^−3^ mol/L	Na^+^	Mg^2+^	Ca^2+^	K^+^	Cl^−^	Alkalinity	${SO}_{4}^{2-}$	${NO}_{3}^{-}$	SiO_2_	pH	DOC (mg/L)
Seine River water	0.429	0.189	2.366	0.077	0.617	3.807	0.334	0.382	0.06	8.05	2.445

**Table 2:** Elemental composition XRF of all the produced ZnS: Mn NPs, deduced by XRF.

NPs		Zn	S	Mn	Mn/(Mn + Zn)_exp_	Mn/(Mn + Zn)_XRF_
ZnS:Mn (0.5%)	mass (μg)	32.315	11.034	0.048		
	%mol	58.9	41.0	0.10	0.005	0.002
ZnS:Mn (2.0%)	mass (μg)	46.842	14.838	0.668		
	%mol	60.1	38.8	1.1	0.020	0.018
ZnS:Mn (4.0%)	mass (μg)	22.464	8.427	0.427		
	%mol	55.9	42.8	1.3	0.040	0.023
ZnS:Mn (10%)	mass (μg)	8.336	3.5	1.420		
	%mol	48.6	41.6	9.8	0.100	0.168

## Data Availability

Data is contained within the article or/and supplementary material.

## Supplementary Materials

The following supporting information can be downloaded at: https://www.mdpi.com/article/10.3390/jox14020028/s1. Table S1: Preparation of stock solutions. Table S2: Preparation of BG11 medium. Table S3: pH of the aqueous media containing various amounts of ZnS:Mn (4.0%). Figure S1: TEM image, size distribution and EDS of (a) ZnS: Mn (2.0%) and (b) ZnS:Mn (4.0%) NPs. Figure S2: TEM image (a) ZnS: Mn (0.5%) and (b) ZnS:Mn (10%) NPs. Figure S3: Size of ZnS:Mn (0.5%, 2.0%, 4.0%) NPs, (a–c) in BG11 and (d–f) in SRW. Figure S4: Size of ZnS:Mn (10%) NPs in Milli-Q water. Figure S5: Zeta potential of (a) ZnS:Mn (0.5%) NPs, (b) ZnS:Mn (2.0%) NPs, and (c) ZnS:Mn (4.0%) NPs. Figure S6: Chlorella vulgaris in the presence of Mn^2+^. Figure S7: PAM and viability of ZnS:Mn (0.5%) NPs. Figure S8: ATP and SOD activity of ZnS:Mn (0.5%) NPs. Figure S9: PAM and viability of ZnS:Mn (2.0%) NPs. Figure S10: ATP and SOD activity of ZnS:Mn (2.0%) NPs. Figure S11: PAM and viability of ZnS:Mn (4.0%) NPs. Figure S12: ATP and SOD activity of ZnS:Mn (4.0%) NPs. Figure S13: Photosynthetic activity of ZnS:Mn (10%) NPs. Figure S14: The Chlorella vulgaris thin sections after exposure to 100 mg L-1 ZnS:Mn (10%) NPs, (a) BG11, and (b) SRW.
