# Supplementary material for: Aquatic Fate and Ecotoxicology Effect of ZnS:Mn Quantum Dots on Chlorella vulgaris in Fresh Water
Source: J Xenobiot. 2024 Mar 26;14(2):467–83. doi: 10.3390/jox14020028 (PMC11036285; doi:10.3390/jox14020028)
Supplement: Supplementary file 1 [file jox-14-00028-s001.zip › jox-2915376-supplementary.pdf]

Aquatic Fate and Ecotoxicology Effect of ZnS:Mn Quantum Dots on *Chlorella vulgaris* in Fresh Water

Toxicity of ZnS:Mn (0.5%) NPs

Photosynthetic activity and viability of *Chlorella vulgaris* are shown in Figure 4. The photosynthetic activity of the algae in the BG11 medium decreased from the very beginning of the 1st day of the test, but then, the algae seemed to recover from the toxic effect until the last test day (after 96 h, i.e. 4 days). Again, we observed that high NPs concentrations (100 mg/L) exhibited higher toxicity than lower NPs concentrations (20 mg/L). In SRW, photosynthetic activity decreased during the first three days of the test, in the presence of NPs in concentrations equal to 20 mg/L and 50 mg/L, while it decreased only slightly in the presence of NPs of concentration equal to 100 mg/L; this may be due to a stronger aggregation of the nanoparticles when the concentration increased.

Figure 4 d-f) showed the viability of *Chlorella vulgaris* in the presence of NPs was clearly reduced in BG11 throughout the test period, while it decreased during the first 48 h of testing in SRW. Finally, we could see that the toxic effect was much stronger for high concentrations of NPs (e.g., 100 mg/L).

Figure 5 shows the intracellular ATP level and SOD activity of Chlorella exposed to ZnS:Mn (0.5%) NPs. In Figure 5 a-c) we observed that the ATP content decreased on the 4th day of the test (i.e., after 72 h) in the BG11 medium, slightly decreased during the first two days of the test in the SRW medium. This confirmed that the toxic effect of ZnS:Mn (0.5%) NPs could affect the activity of mitochondria. In Figure 5 d-f), we observed that SOD activity increased to varying degrees over the test period. For low concentrations of NPs, SOD activity increased little, even for a concentration of 100 mg/L, which was probably due to the aggregation of NPs. ROS production increased only on the last day of testing in SRW medium. The SOD activity was higher in the groups exposed to concentrations of 100 mg/L of NP than in the other groups. Therefore, we clearly see that ZnS:Mn (0.5%) NPs could induce the toxic effect on *Chlorella vulgaris* and cause cell death in all media. The NPs introduced in higher concentration were more toxic, in the BG11. NPs could induce ROS production in *Chlorella vulgaris* cells present in SRW as well as a toxic effect on cellular metabolic activity; it represents one of the main impacts of NP toxicity.

Toxicity of ZnS: Mn (2.0%)

The toxicity of ZnS:Mn (2.0%) NPs was then also studied on *Chlorella vulgaris*, by introducing 20 mg/L, 50 mg/L and 100 mg/L NPs in culture flasks containing 60 ml of algae culture. Figure 6 presents the results of fluorimetry (PAM) as well as the viability of the algae in the presence of ZnS: Mn (2.0%) NPs.

In BG11, photosynthetic activity decreased compared to the control group throughout the test period (Figure 6 a-c). Moreover, the photosynthetic activity of microalgae in contact with

the concentration of 100 mg/L of NPs was much more influenced than the other research groups. In the SRW, the photosynthetic activity was only slightly reduced on the 4th day of the test, even in the presence of high levels of NPs.

The viability of *Chlorella vulgaris* (Figure 6 d-f) decreased significantly during the first two days of testing in BG11 medium treated with 100 mg/L of NPs. The toxic effect continued throughout the duration of the test since even the viability of the groups exposed to the lowest concentration decreased slightly 48 hours later. While the decrease in viability only appeared on the first day of the test in the SRW containing high levels of NPs, the viability of *Chlorella vulgaris* in the medium containing 20 mg/L of NPs does not seem to be affected.

Figure 7 shows the intracellular ATP content and SOD activity of *Chlorella vulgaris* in media exposed to ZnS:Mn (2.0%) NPs. In Figure 7 a-c) we observed that the intracellular ATP content decreased in the BG11 medium from the first day of the test, but the toxic effect of the NPs on the mitochondria was much higher after 48 h. The activity of *Chlorella vulgaris* mitochondria in the SRW in the presence of 100 mg/L of NPs was more affected than in the other two research groups, while the microalgae exposed to 20 mg/L of NPs did not seem affected. Moreover, in BG11 and SRW, the SOD activity of *Chlorella vulgaris* (Figure 7 d-f), increased slightly during the first two days of testing, and more markedly 48 h later. Above all, we could conclude that ZnS:Mn NPs (2.0%) induced ROS production after 48 hours of testing in BG11 and SRW media. The ROS produced may have a severe effect on the mitochondria from the third day of testing (intracellular ATP levels) and then indirectly affect other metabolic activities, such as photosynthetic activity and viability.

Toxicity of ZnS: Mn (4.0%)

The toxic effect of ZnS:Mn (4.0%) NPs on *Chlorella vulgaris* was also evaluated (Figure 8). The photosynthetic activity of *Chlorella vulgaris* decreased immediately from the very first day of testing to the last day compared to the control group, and the toxic effect increased with the increasing of the concentration of NPs used. *Chlorella vulgaris* showed lower photosynthetic activity throughout the test period in contact with 100 mg/L of NPs. In the SRW, the photosynthetic activity did not seem to be affected by the high concentrations (50 mg/L and 100 mg/L) of ZnS:Mn (4.0%) NPs, while in the BG11 containing 20 mg/L, the photosynthetic activity significantly decreased throughout the duration of the test; this was probably because NPs, in high concentrations, were much easier to aggregate, and therefore more difficult for *Chlorella vulgaris* to absorb them. DLS measurements supported the aggregation of NPs in SRW (size greater than 700 nm at pH 8, the pH of the algae culture has been shown in Table 5). In Figure 8 d-f), algae viability decreased slightly during the first two days of testing in BG11 and SRW media within 24 h, again in the presence of a high concentration of ZnS:Mn (4.0%) (100 mg/L of NPs).

Then, intracellular ATP content and SOD activity were measured to study the effect of ROS production on mitochondrial activity. In Figure 9 a-c), one can see that the intracellular ATP content decreased to varying degrees in BG11 and SSW while it just decreased slightly at 3 h and then 72 h in SRW, which corresponds to the photosynthetic activity and the viability of

Chlorella vulgaris. It is also clear that a high NP content (100 mg/L) is more toxic. In Figure 9 d-f), one can notice that the SOD increased in the BG11 and SRW media from the first day of testing to the fourth day of testing, and that it decreased after 96h. Therefore, we could confirm that NPs may induce the production of ROS, which may have a toxic effect on photosynthetic activity, cell viability and other metabolic activities.

Blue-Green medium (BG11)

According to the recipe Sendersky & al, bio-protocol, 2017 with three main changed for the buffer concentration, the pH, the copper concentration (modified by Claude Yepremian, MNHN, 2020)

N° solution Stock	Component	Mass (g) for 200 mL	Molar concentration	Notes
1.	$MgSO_4.7H_2O$	1.3	0.284 mM	Don’t autoclave!
	$CaCl_2.2H_2O$	0.72	0.245 mM	
2.	$K_2HPO_4.3H_2O$	0.80	0.175 mM	Storage 3 months at 4 ℃
	EDTA (Na_2)	0.02	2.26 μM	
3.	Citric Acid	0.12	31.2 μM	Storage 10 days in the dark
	Ammonium Ferric Citrate Green	0.12	30.0 μM	
4. Metal trace solution	H_3BO_3	572	46.3 μM	Storage 2 years at 4 ℃
	$MnCl_2.4H_2O$	362	9.15 μM	
	$ZnSO_4.7H_2O$	44	0.77 μM	
	$Na_2MoO_4.2H_2O$	78	1.61 μM	
	$CuSO_4.5H_2O$	4	0.08 μM	
	$CO(NO_3)2.6H_2O$	10	0.17 μM	
5. Vitamin solution (optional)	Cyanocobalamin (B12)	8		Vitamin solution n° 7 of JM/JM2 media, vitamins are dissolved in ultrapure Water pH=4
	Thiamine HCl (B1)	8		
	Biotin	8		

Table S1. Preparation of stock solutions.

For the 5 stock solutions: Adjust to 200 mL with ultrapure water

For the stock solution 2, 3, 4, 5: Autoclave at 121 ℃ for 20 minutes

Preparation of BG11 medium with stock solutions:

(1) Add 950 mL ultrapure water in 1 L bottle,

(2) Add 1.5 g of $NaNO_3$ (17.6 mM)

(3) Add the stock solution according to the table 4.

N° solution stock	Volume (mL) for 1 L
1	10
2	10
3	10
Metal trace solution (4)	1
Vitamin solution (5)	1
HEPES (5 mM)	1.19 g

Table S2. Preparation of BG11 medium

Adjust pH=7.30 with ≈2 mL NaOH (1 M)

Adjust the volume to 1 L with ultrapure water

Autoclave at 121 °C for 15-20 minutes

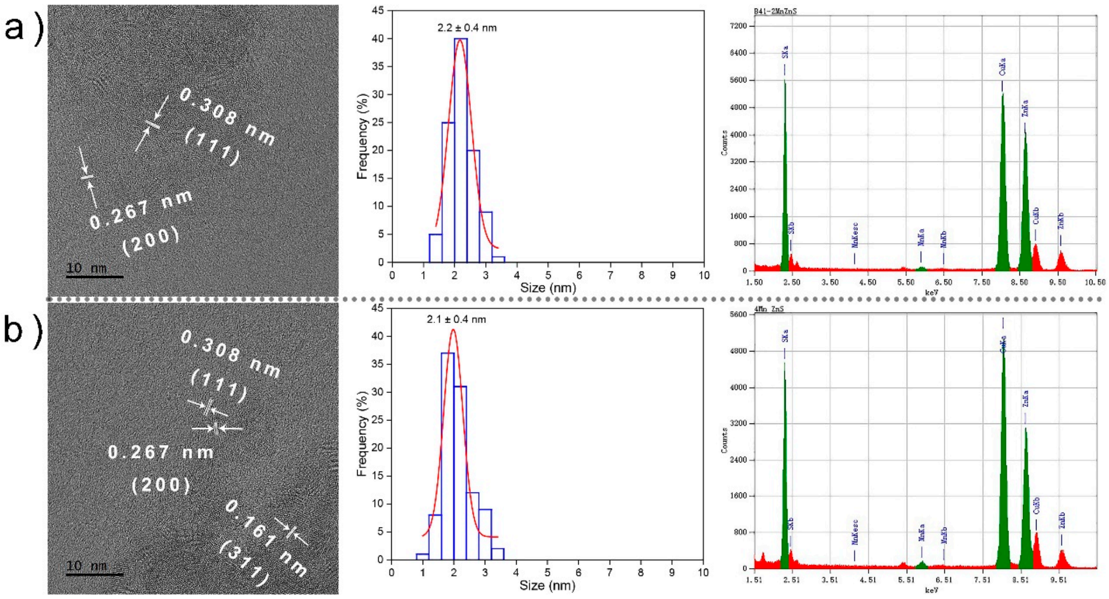

Figure S1. TEM image, size distribution and EDS of a) ZnS: Mn (2.0%) and b) ZnS:Mn (4.0%) NPs

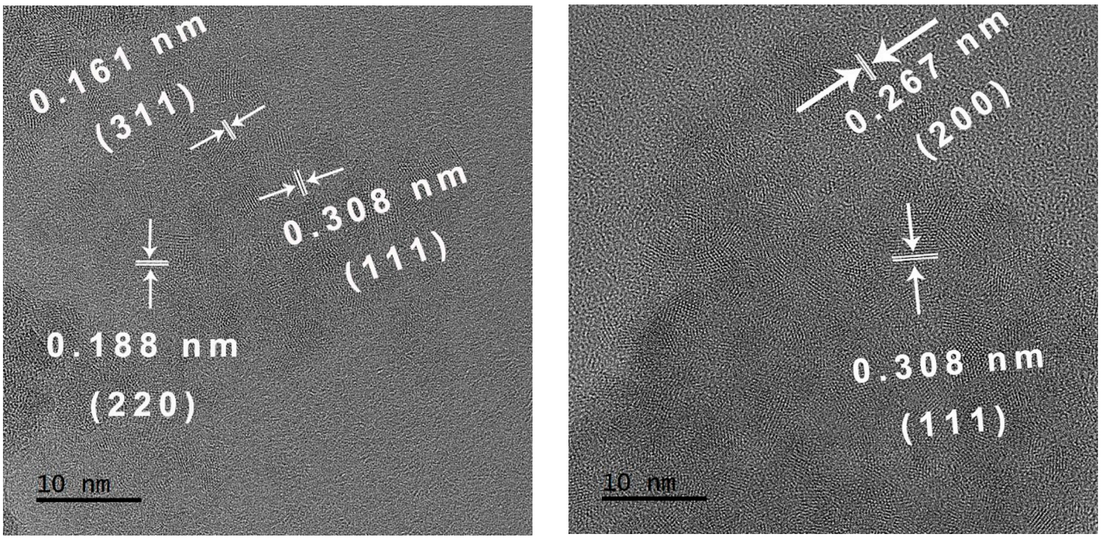

Figure S2. TEM image a) ZnS: Mn (0.5%) and b) ZnS:Mn (10%) NPs

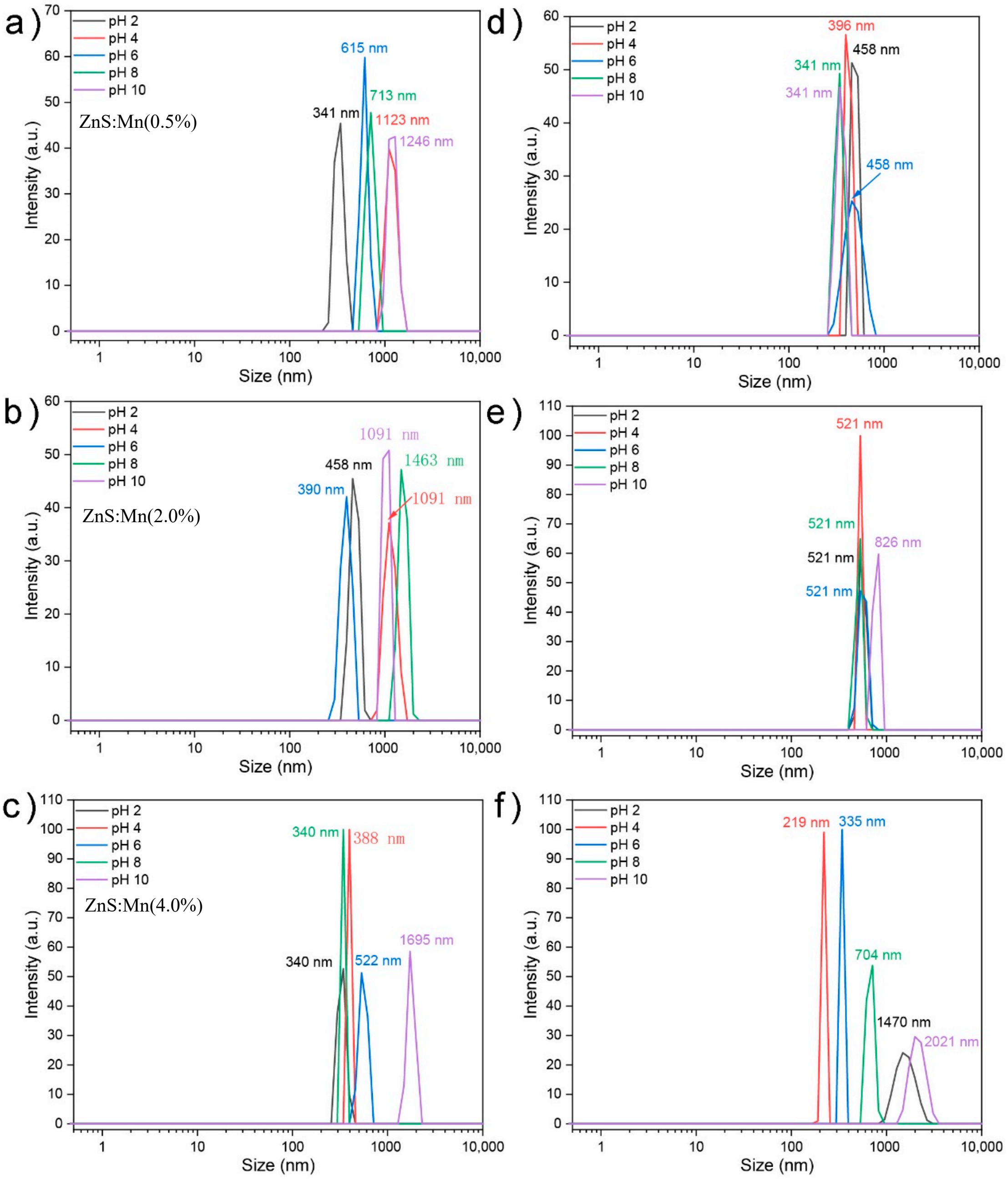

Figure S3. Size of ZnS:Mn (0.5%, 2.0%, 4.0%) NPs, (a-c) in BG11, (d-f) in SRW

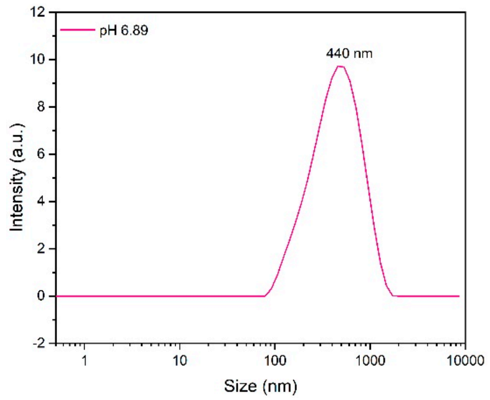

Fig S4. Size of ZnS:Mn (10%) NPs in Milli-Q water

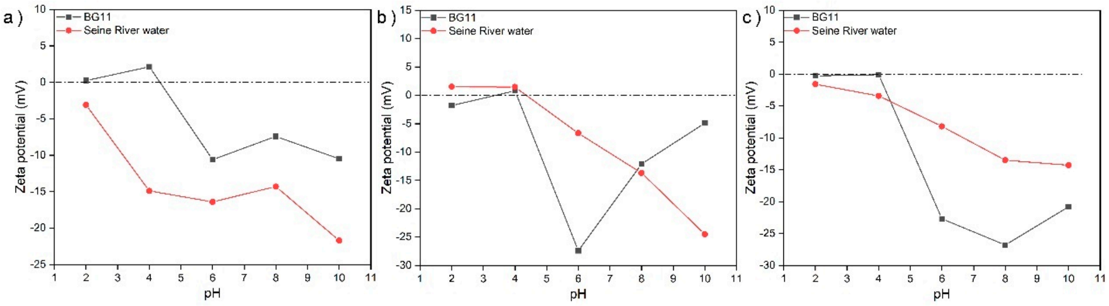

Figure S5. Zeta potential of a) ZnS:Mn (0.5%) NPs b) ZnS:Mn (2.0%) NPs and c) ZnS:Mn (4.0%) NPs

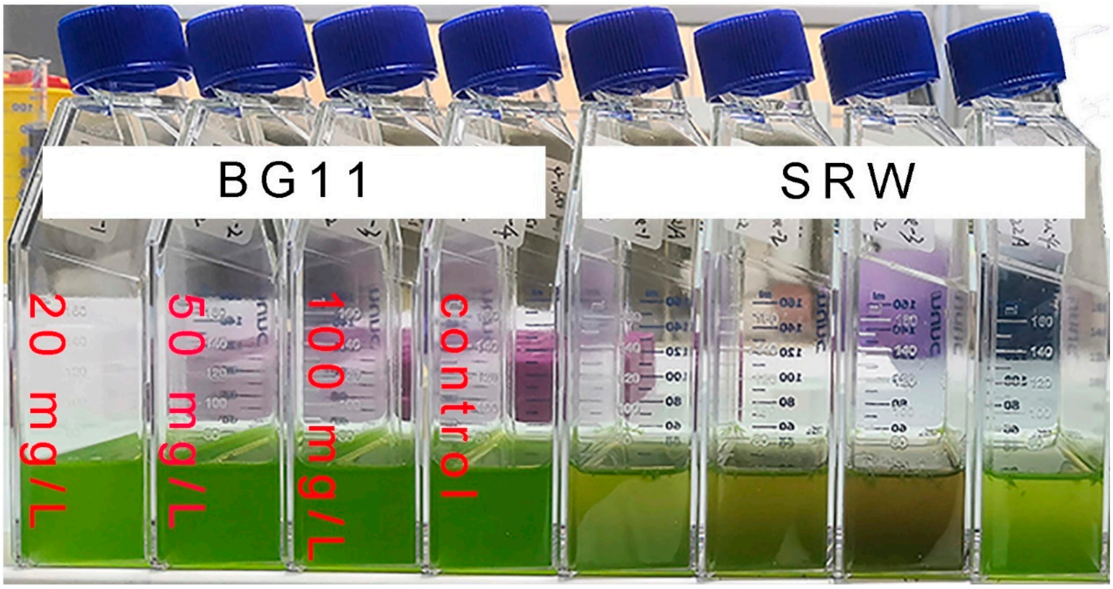

Figure S6. *Chlorella vulgaris* in the presence of Mn^{2+}

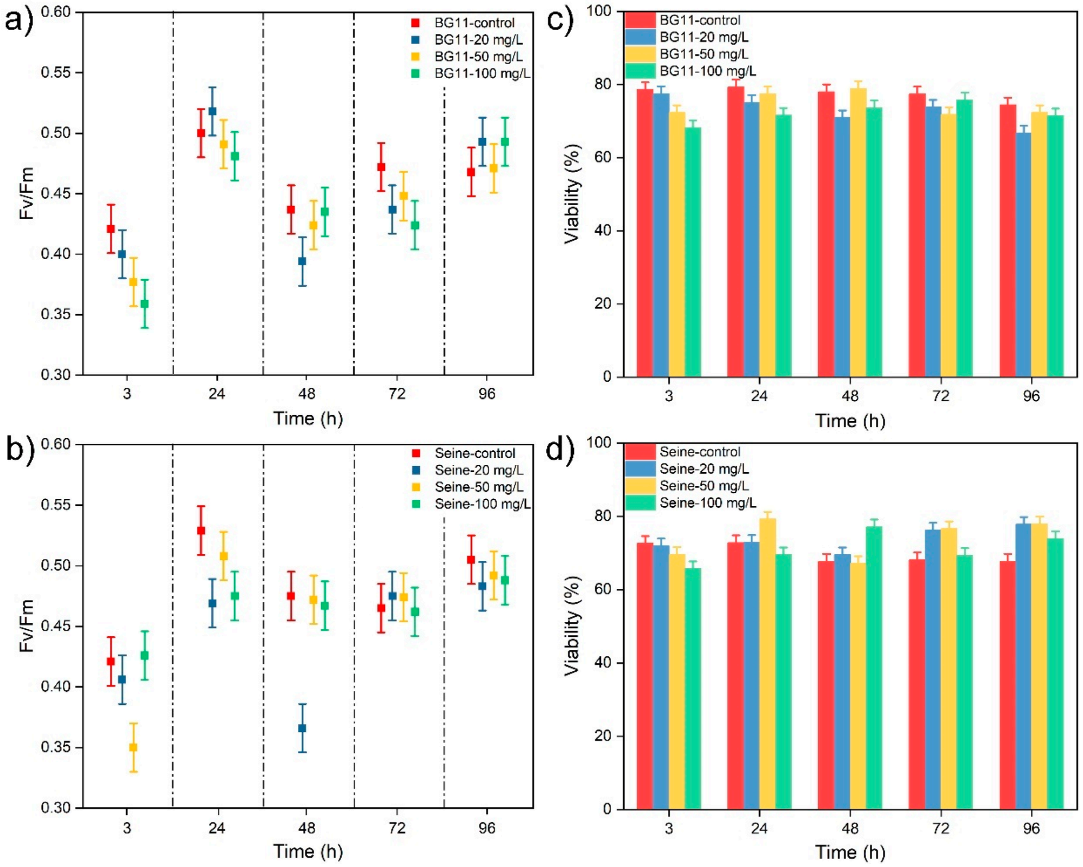

Figure S7. PAM and viability of ZnS:Mn (0.5%) NPs

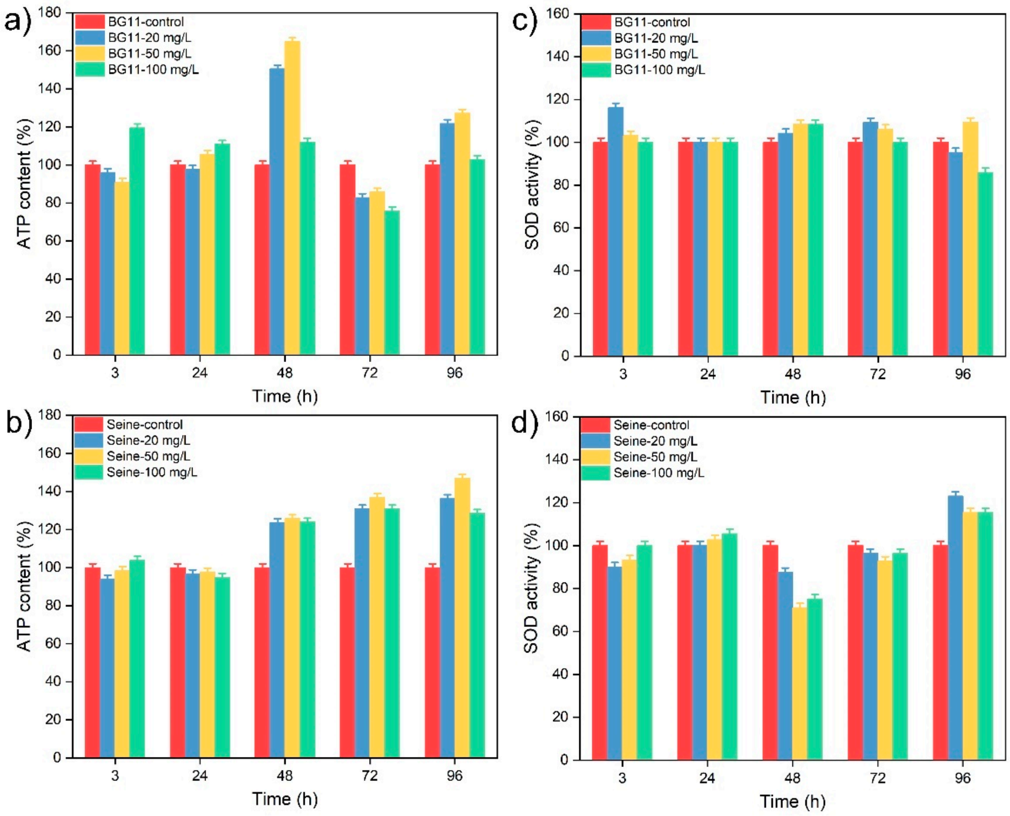

Figure S8. ATP and SOD activity of ZnS:Mn (0.5%) NPs

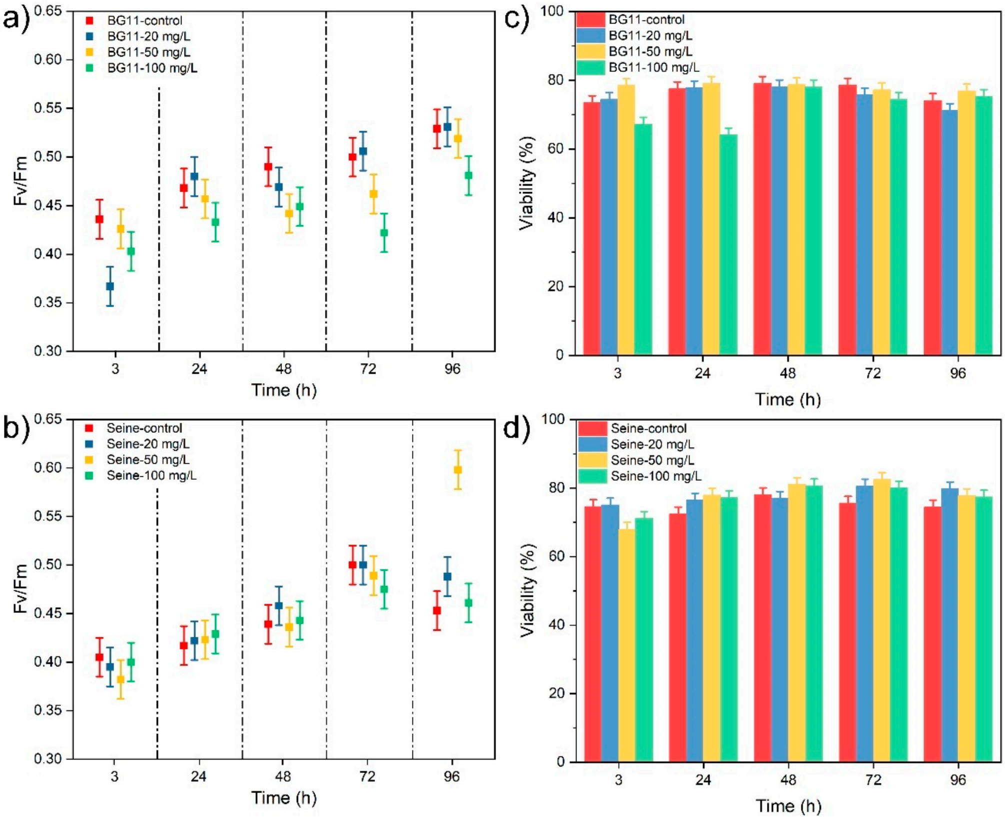

Figure S9. PAM and viability of ZnS:Mn (2.0%) NPs

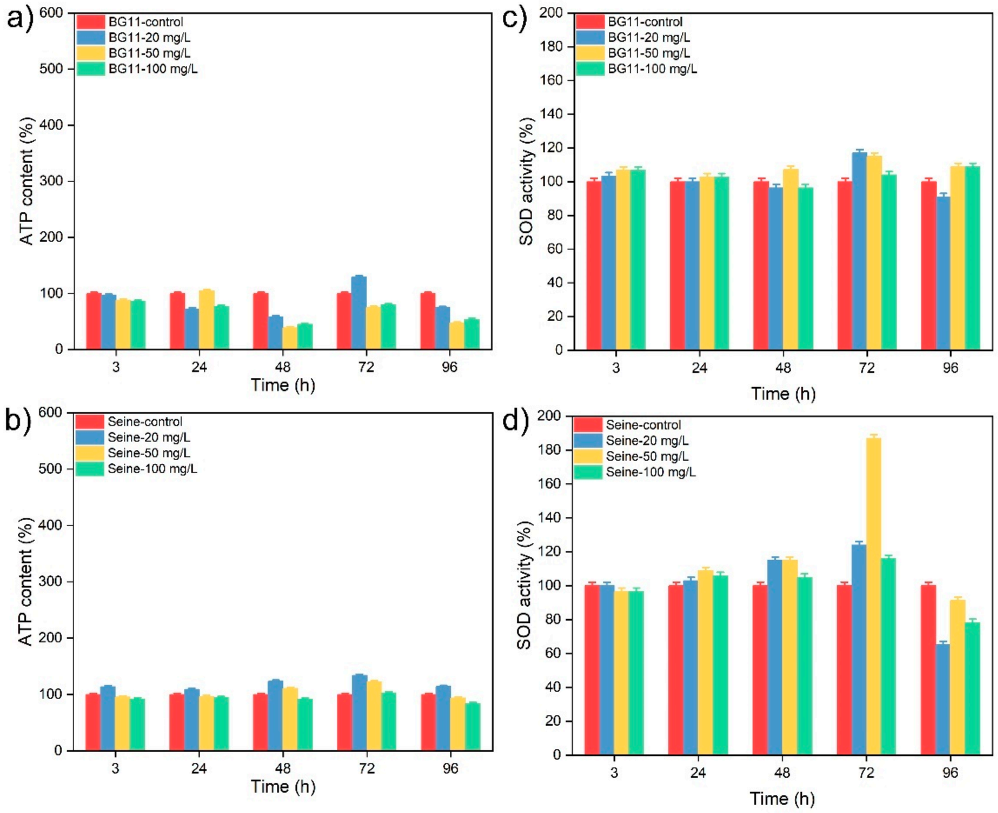

Figure S10. ATP and SOD activity of ZnS:Mn (2.0%) NPs

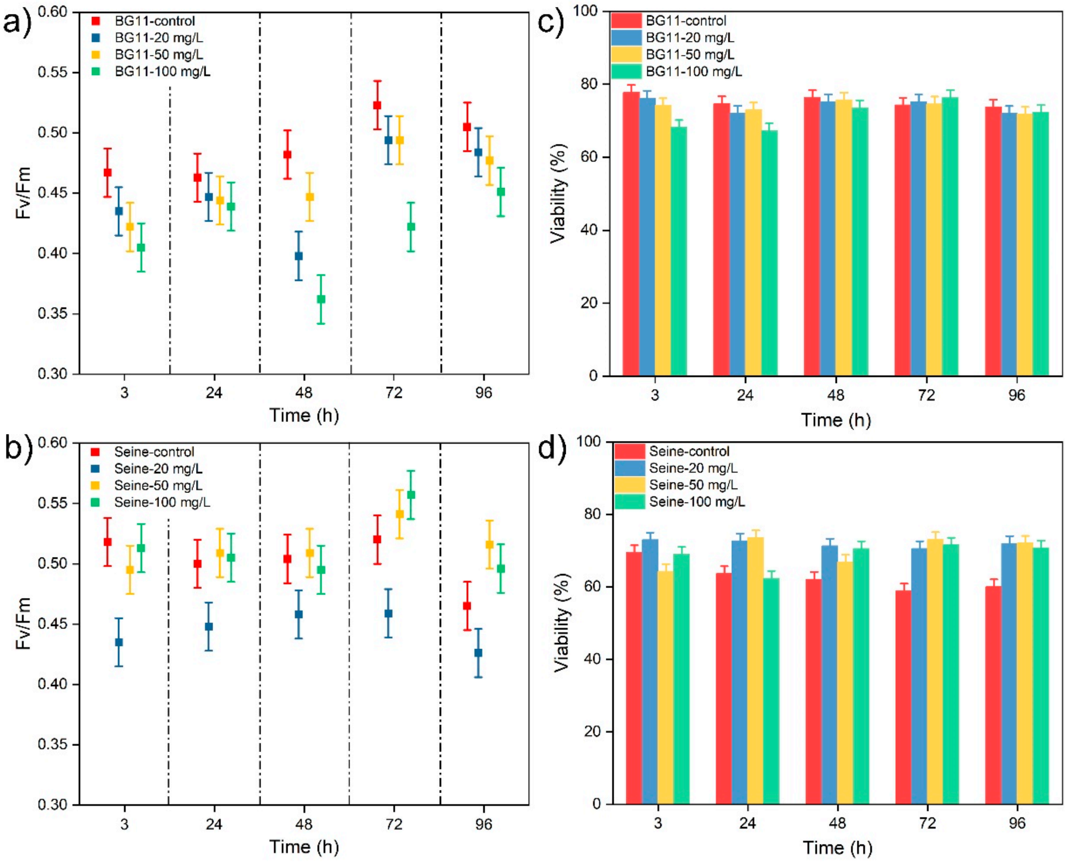

Figure S11. PAM and viability of ZnS:Mn (4.0%) NPs

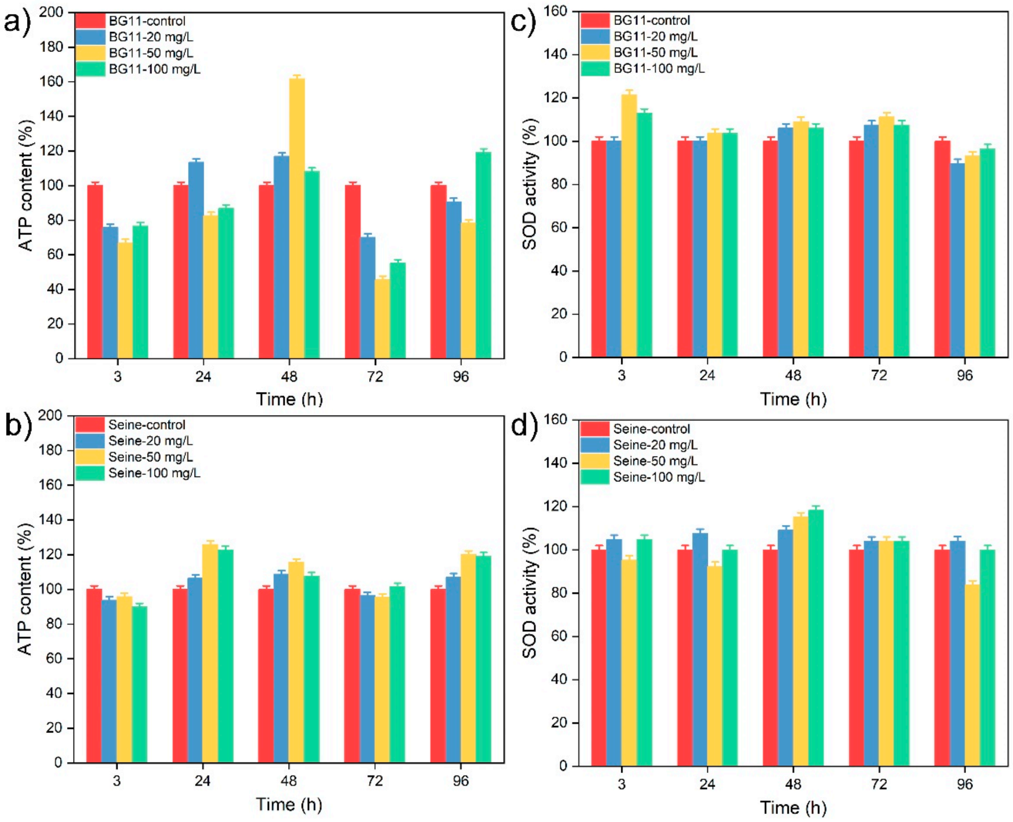

Figure S12. ATP and SOD activity of ZnS:Mn (4.0%) NPs

Table S3. pH of the aqueous media containing various amounts of ZnS:Mn (4.0%)

Medium	BG11				SRW			
	20 mg/L	50 mg/L	100 mg/L	control	20 mg/L	50 mg/L	100 mg/L	control
pH	7.60	7.54	7.36	8.12	8.68	8.65	8.69	9.34

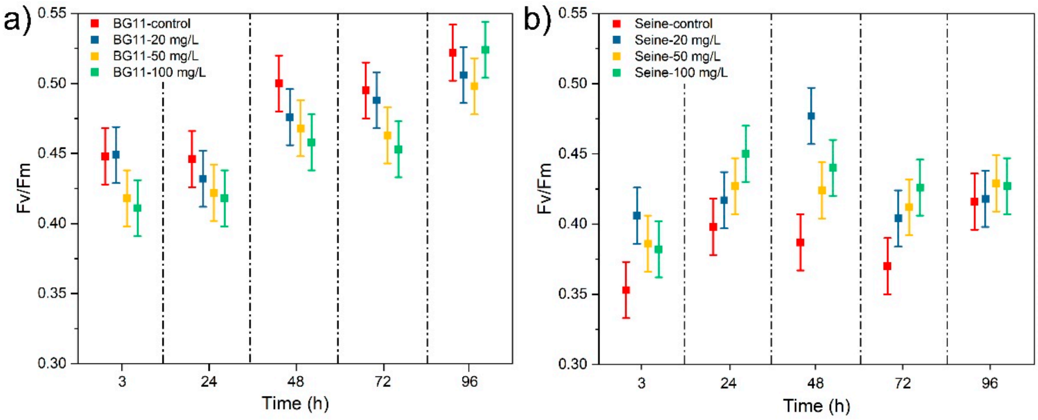

Figure S13. Photosynthetic activity of ZnS:Mn (10%) NPs

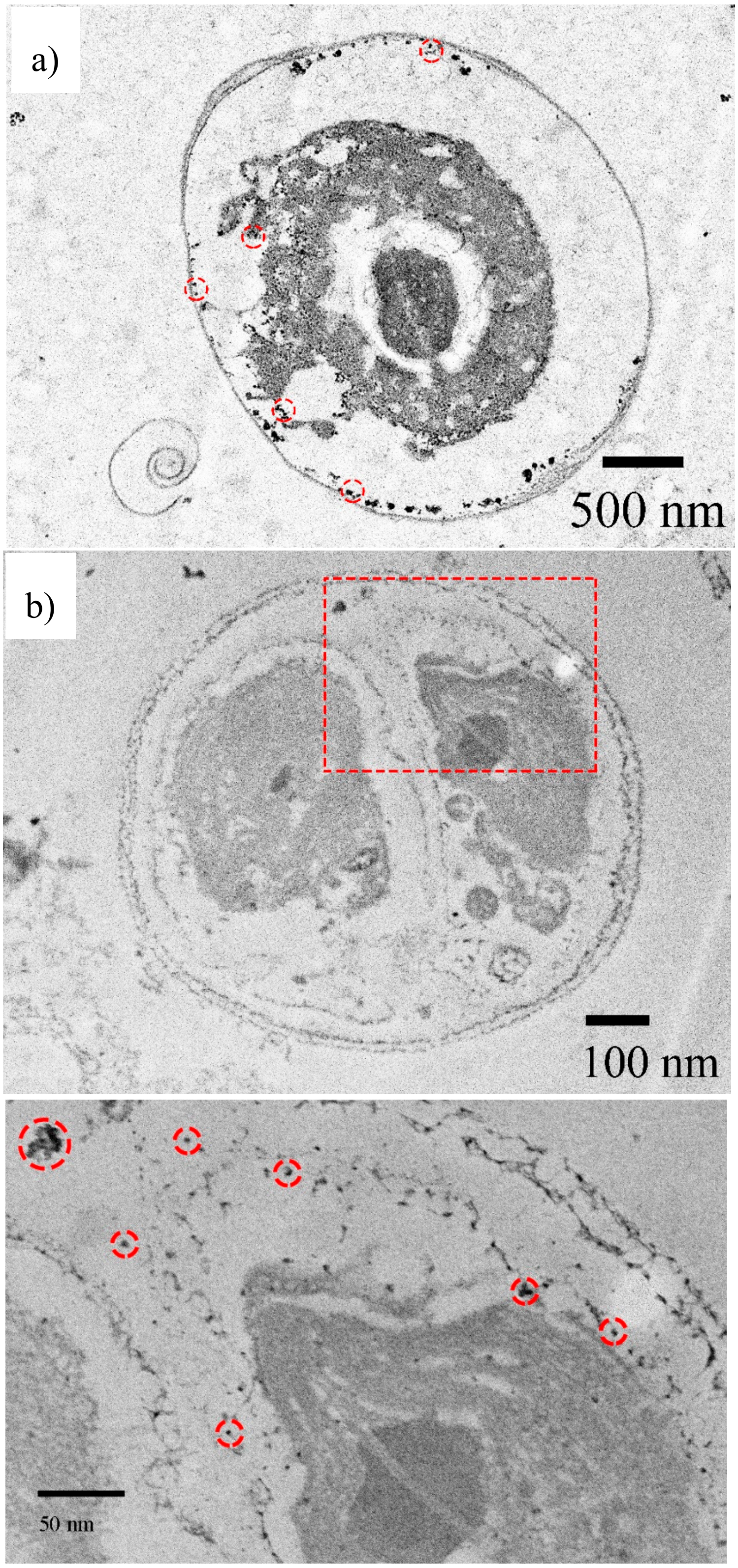

Figure S14. The *Chlorella vulgaris* thin sections after exposure to 100 mg L^{-1} ZnS:Mn (10%) NPs, (a) BG11, (b) SRW
